# Correction: Genomic architecture and evolutionary antagonism drive allelic expression bias in the social supergene of red fire ants

**DOI:** 10.7554/eLife.64678

**Published:** 2020-11-09

**Authors:** Carlos Martinez-Ruiz, Rodrigo Pracana, Eckart Stolle, Carolina Ivon Paris, Richard A Nichols, Yannick Wurm

Martinez-Ruiz C, Pracana R, Stolle E, Paris CI, Nichols RA, Wurm Y. 2020. Genomic architecture and evolutionary antagonism drive allelic expression bias in the social supergene of red fire ants. *eLife* **9**:e55862. doi: 10.7554/eLife.55862.

Published 10, August 2020

After publication, we noticed minor typographical errors that were missed during proofing in the captions of Figures 3 and 4d and Figure 1 Supplementary figures 2, 3 and 4 as well as Results and Methods. All of these errors emerged due to text re-arrangements in the later stages of review or during typesetting. None of the errors affect our results nor our interpretations.

Original caption of Figure 3:

Values of P_B_ below 0.5 therefore indicate higher expression in SB/SB queens from single-queen colonies; values above 0.5 indicate higher expression in SB/Sb queens from multiple-queen colonies. Values of P_MQ_ above 0.5 indicate allelic bias towards SB; values below 0.5 indicate bias towards Sb.

Corrected caption of Figure 3:

Values of P_MQ_ below 0.5 therefore indicate higher expression in SB/SB queens from single-queen colonies; values above 0.5 indicate higher expression in SB/Sb queens from multiple-queen colonies. Values of P_B_ above 0.5 indicate allelic bias towards SB; values below 0.5 indicate bias towards Sb.

Original caption of Figure 4d:

...the genes with higher expression of the Sb allele (x > 0) tend to be more highly expressed in queens from multiple-queen colonies (y < 0)...

Corrected caption of Figure 4d:

...the genes with higher expression of the Sb allele (y < 0) tend to be more highly expressed in queens from multiple-queen colonies (x > 0)...

Original caption of Figure 1 Figure supplements 2, 3 and 4

Differences in allele-specific expression between variants (x axis) for all genes in the supergene with enough expression information (y axis). Significant expression differences (BH adjusted p<0.05 from Wald test in DESeq2) are in green and the RefSeq ID of the significant gene is outlined in the y axis.

Corrected caption of Figure 1 Figure supplements 2, 3 and 4

Differences in allele-specific expression between variants (y axis) for all genes in the supergene with enough expression information (x axis). Significant expression differences (BH adjusted p<0.05 from Wald test in DESeq2) are in green and the RefSeq ID of the significant gene is outlined in the x axis.

In Results:

Original:

Most of these genes (Bergero and Charlesworth, 2009) had higher expression in SB...

Corrected:

Most of these genes (12) had higher expression in SB...

In Methods:

Original:

We then aligned the RNAseq reads from each sample to the regular reference genome (version gnG; RefSeq GCF_000188075.1; [Darlington and Mather, 1949])...

Corrected:

We then aligned the RNAseq reads from each sample to the regular reference genome (version gnG; RefSeq GCF_000188075.1)...

Original:

For the North American and Taiwanese datasets (Wurm et al., 2011),...

Corrected:

For the North American and Taiwanese datasets (Wurm et al., 2011; Fontana et al., 2020),...

Original:

...single-queen vs. multiple-queen expression for each gene (X_SQ_ vs. X_MQ_) from the Wurm et al., 2011 dataset and expression of the SB allelic variant vs. the Sb (X_B_ vs. X_b_) within each gene from the Morandin et al., 2016...

Corrected:

...single-queen vs. multiple-queen expression for each gene (X_SQ_ vs. X_MQ_) from the Morandin et al., 2016 dataset and expression of the SB allelic variant vs. the Sb (X_B_ vs. X_b_) within each gene from the Wurm et al., 2011...

The article has been corrected accordingly.

