# Supplementary material for: Genomic architecture and evolutionary antagonism drive allelic expression bias in the social supergene of red fire ants
Source: eLife. 2020 Aug 10;9:e55862. doi: 10.7554/eLife.55862 (PMC7476760; doi:10.7554/eLife.55862)
Supplement: Transparent reporting form [file elife-55862-transrepform.docx]

***eLife’s* transparent reporting form**

We encourage authors to provide detailed information *within their submission* to facilitate the interpretation and replication of experiments. Authors can upload supporting documentation to indicate the use of appropriate reporting guidelines for health-related research (see [EQUATOR Network](http://www.equator-network.org/%20)), life science research (see the [BioSharing Information Resource](https://biosharing.org/)), or the [ARRIVE guidelines](http://www.plosbiology.org/article/info:doi/10.1371/journal.pbio.1000412) for reporting work involving animal research. Where applicable, authors should refer to any relevant reporting standards documents in this form.

If you have any questions, please consult our Journal Policies and/or contact us: [editorial@elifesciences.org](mailto:editorial@elifesciences.org).

**Sample-size estimation**

- You should state whether an appropriate sample size was computed when the study was being designed
- You should state the statistical method of sample size computation and any required assumptions
- If no explicit power analysis was used, you should describe how you decided what sample (replicate) size (number) to use

Please outline where this information can be found within the submission (e.g., sections or figure legends), or explain why this information doesn’t apply to your submission:

We performed power analyses to ensure that the conclusions reached based on both our DNA and RNA-seq datasets were based on a robust enough sample size. This information can be found in the sections “Identifying SNPs with fixed differences between SB and Sb males” and “Estimating read counts from alternate social chromosome variants in heterozygous individuals” in Methods.

The sample sizes were based on availability of material, established practice for sequencing-based studies, and sequencing feasibility (e.g., obtaining high quality RNA from samples from Argentina or Brazil that match social form, species and genotype criteria is complex).

**Replicates**

- You should report how often each experiment was performed
- You should include a definition of biological versus technical replication
- The data obtained should be provided and sufficient information should be provided to indicate the number of independent biological and/or technical replicates
- If you encountered any outliers, you should describe how these were handled
- Criteria for exclusion/inclusion of data should be clearly stated
- High-throughput sequence data should be uploaded before submission, with a private link for reviewers provided (these are available from both GEO and ArrayExpress)

Please outline where this information can be found within the submission (e.g., sections or figure legends), or explain why this information doesn’t apply to your submission:

The information on replicates for the newly generated and publicly available RNA-seq data is in the section “RNA sequencing of fire ants” from methods. For the DNA data, we detail the replicates used for each population in the section “Identifying SNPs with fixed differences between SB and Sb males”. Additionally, we provide further details in tables S5 and S6.

Specifically, we generated 24 whole transcriptome RNAseq libraries (from across 4 sample types; i.e. 6 replicates per sample, with each replicate from a different biological entity (colony)). We obtained 14.8 million read pairs on average per sample. We added to this 14 samples from other studies.

We generated 36 whole genome DNAseq libraries, with an average of 17.8 milllion read pairs per sample, and combined these with 14 previously published DNAseq libraries. This provided 10.2x average genome coverage per sample. We specifically used PCR-free TruSeq library preparation protocols to obtain homogeneous coverage. Because male ants are haploid, this provides plenty of power to appropriately call genotypes.

**Statistical reporting**

- Statistical analysis methods should be described and justified
- Raw data should be presented in figures whenever informative to do so (typically when N per group is less than 10)
- For each experiment, you should identify the statistical tests used, exact values of N, definitions of center, methods of multiple test correction, and dispersion and precision measures (e.g., mean, median, SD, SEM, confidence intervals; and, for the major substantive results, a measure of effect size (e.g., Pearson's r, Cohen's d)
- Report exact p-values wherever possible alongside the summary statistics and 95% confidence intervals. These should be reported for all key questions and not only when the p-value is less than 0.05.

Please outline where this information can be found within the submission (e.g., sections or figure legends), or explain why this information doesn’t apply to your submission:

All the statistical tests and models used in this study are reported in the Methods section. Additionally, we report exact statistics and p values (where available) throughout the Results section. All figures include the exact values of N and p values (<0.05 and otherwise).

(For large datasets, or papers with a very large number of statistical tests, you may upload a single table file with tests, Ns, etc., with reference to sections in the manuscript.)

**Group allocation**

- Indicate how samples were allocated into experimental groups (in the case of clinical studies, please specify allocation to treatment method); if randomization was used, please also state if restricted randomization was applied
- Indicate if masking was used during group allocation, data collection and/or data analysis

Please outline where this information can be found within the submission (e.g., sections or figure legends), or explain why this information doesn’t apply to your submission:

The details of RNA and DNA extraction methods to avoid batch effects are detailed in the sections “RNA sequencing of fire ants” and “Identifying SNPs with fixed differences between SB and Sb males” in Methods.

Sampling was done randomly and blindly to study group. The same was true for all lab work as well as dissections. Among possible samples, we chose a geographically balanced representation to avoid introducing any biases.

**Additional data files (“source data”)**

- We encourage you to upload relevant additional data files, such as numerical data that are represented as a graph in a figure, or as a summary table
- Where provided, these should be in the most useful format, and they can be uploaded as “Source data” files linked to a main figure or table
- Include model definition files including the full list of parameters used
- Include code used for data analysis (e.g., R, MatLab)
- Avoid stating that data files are “available upon request”

Please indicate the figures or tables for which source data files have been provided:

All figures and tables are based on RNA or DNA sequencing data that is either already publicly available or will be publicly available at moment of publication. All the code used for the analyses and the figures in this study will be made available at <http://github.com/MartinezRuiz-Carlos/2019-11_allele_specific_expression_fire_ant> and is available during review at <http://bit.ly/ase_code>
